# “We are ambassadors, we are advocates”: rare disease patient advocacy groups as knowledge brokers across health and social systems—a qualitative study from Poland

**DOI:** 10.3389/fpubh.2026.1743598

**Published:** 2026-02-26

**Authors:** Jan Domaradzki

**Affiliations:** Department of Social Sciences and Humanities, Poznan University of Medical Sciences, Poznań, Poland

**Keywords:** advocacy, awareness-raising, education, experiential knowledge, knowledge brokerage, patient advocacy groups, public engagement, rare disease patient advocacy groups

## Abstract

**Background:**

Rare diseases (RDs) pose a significant public health concern, particularly in Poland, where awareness among healthcare professionals, educators, and the general public remains low. Rare disease patient advocacy groups (RDPAGs), often led by caregivers, play a vital yet under recognized role in addressing these gaps through informal education and advocacy. This study explores how RDPAGs help fill systemic gaps in RD-related education.

**Methods:**

This qualitative descriptive study draws on 11 interviews with leaders of Polish RDPAGs engaged in advocacy, education, and public engagement. Interviews were analysed using Reflexive Thematic Analysis, following Braun and Clarke’s six-phase framework, and reported in line with COREQ guidelines.

**Results:**

The analysis identified four interrelated themes describing the educational and advocacy practices of RDPAGs. Participants portrayed RDPAGs as: (1) acting as ambassadors of knowledge across sectors by translating scientific and experiential expertise into accessible information; (2) supporting families through lived experience by providing curated resources, peer mentoring, and practical guidance, particularly following diagnosis; (3) engaging with and sometimes challenging professional expertise by sharing caregiver-informed knowledge, co-developing materials, and initiating dialogue with healthcare and education professionals; and (4) raising public awareness and engaging in policy-oriented advocacy through campaigns and inter-organizational collaboration. Collectively, participants described these practices as addressing systemic knowledge gaps and supporting cross-sectoral care navigation.

**Conclusion:**

The findings indicate that healthcare, education, and policy systems in Poland already rely substantially on the informal educational and advocacy work of RDPAGs. Formal recognition and support may help stabilize these contributions, provided that issues of sustainability, role boundaries, and uneven organizational burdens are taken into account.

## 1 Introduction

In the European Union (EU), a rare disease (RD) is defined as a condition affecting fewer than 1 in 2,000 individuals (1). Some conditions are even rarer: ultra-rare diseases affect fewer than 1 in 100,000 people, while hyper-rare conditions impact fewer than 1 in 1,000,000 (2, 3). Recent estimates suggest that over 10,000 RDs have been identified (4), affecting approximately 30 million people in the EU and between 2.5 and 3 million individuals in Poland (5). Although RDs vary widely in etiology, symptoms, and prognosis, approximately 80% are genetic in origin, nearly two-thirds are associated with severe health complications, and around 95% currently lack approved treatment options (6–8). Children account for about half of those affected, with an estimated 30% dying before the age of five (6). As a result, RDs place considerable physical, emotional, and practical burdens on families, who must often navigate fragmented and highly complex care systems over long periods of time (9–12). The rarity and heterogeneity of these conditions amplify systemic challenges and make RDs a significant public health concern (13, 14).

A persistent barrier in the RD field is limited awareness and knowledge among healthcare professionals, policymakers, and the general public. Numerous studies document substantial knowledge gaps among physicians, particularly primary care providers (PCPs), who report inadequate training and low confidence in caring for RD patients (15–21). Given that PCPs encounter RDs in only a small proportion of clinical visits (approximately 1.6%) (22), such limited preparedness is not unexpected. Comparable deficits have also been reported among medical students and nurses, many of whom feel insufficiently prepared to support RD patients (16, 19, 21). This professional knowledge gap is mirrored by superficial public understanding, which undermines institutional prioritization, funding, and coordinated support for affected families (23).

These knowledge deficits contribute to the so-called “diagnostic odyssey,” a prolonged and distressing search for an accurate diagnosis, often involving multiple providers, misdiagnoses, and delayed access to specialized and coordinated care (12, 24, 25). Such delays are associated with inappropriate treatments, avoidable hospitalizations, deterioration in health status, and substantial emotional and practical burdens on caregivers (26). These challenges are further compounded by experiences of stigma and marginalization (27), as well as uneven access to genetic testing, counselling, innovative therapies, and psychosocial or institutional support, which often forces families to bridge systemic gaps through private resources (28–30). At the same time, persistent deficits in genetic and health literacy among both healthcare professionals and laypeople, despite the predominantly genetic nature of most RDs, impair communication, delay intervention, and complicate care planning and outcomes (31, 32). As a result, families frequently assume responsibility for information-seeking, relying on online sources and peer networks to compensate for gaps in formal support (25, 33, 34).

Knowledge deficits are also evident in the education sector. Although many teachers are familiar with general principles of inclusion, they often lack condition-specific knowledge and training required to support students with RDs (35, 36). Limited understanding of symptoms, educational needs, and appropriate accommodations can hinder effective support and inclusion in school settings. This underscores the need for more integrated educational approaches that foster collaboration between teachers, healthcare professionals, and families to ensure appropriate learning conditions for children with RDs (36, 37). Consequently, experts increasingly call for comprehensive education strategies addressing healthcare professionals, patients, and the wider public (38).

In this context, rare disease patient advocacy groups (RDPAGs) represent a prominent site where educational and awareness-raising practices are articulated and enacted in response to perceived systemic knowledge gaps. These organizations have been described as contributing to knowledge circulation, family support, and engagement with healthcare professionals and policymakers through educational initiatives and advocacy efforts (39–41). Their activities operate across multiple levels simultaneously—individual, professional, and institutional—positioning RDPAGs within a shared ecosystem of learning and advocacy.

Importantly, many RDPAG leaders are themselves parents or patients, meaning that lived experience is intrinsically embedded in their educational and advocacy practices. This interweaving of experiential and biomedical knowledge underpins the multidimensional nature of their work and provides a basis for examining their educational roles as a set of interconnected practices rather than as isolated or sector-specific activities.

In Poland, RDPAGs collaborate through support networks, national federations, and umbrella organizations such as the Federation of Polish Patients (FPP) and EURORDIS. As highlighted in the 2025 ORPHAN Report, they play a growing role in disseminating accessible disease-specific information and organizing educational initiatives for both families and professionals (42). Despite this increasing activity, the educational and informational functions of RDPAGs in the Polish context remain largely underexplored in academic research. This gap is particularly notable given their position at the intersection of patient experience, professional communication, and public awareness, where they may function as informal knowledge brokers and agents of change.

From a sociological perspective, these developments resonate with broader debates on lay expertise and the shifting boundaries between professional and experiential knowledge. Research in medical sociology and science and technology studies has shown that lay actors increasingly mobilize experience-based and condition-specific knowledge in ways that reconfigure established boundaries of expertise (43–45). In the field of genetics in particular, patient groups have been described as forming new modes of “genetic” or “biological citizenship,” whereby individuals and collectives claim epistemic authority and moral legitimacy based on embodied experience and shared biological conditions (46). Rather than assuming the legitimacy or effectiveness of such forms of knowledge *a priori*, this study approaches RDPAGs as empirical sites where lay expertise is articulated, negotiated, and enacted in relation to professional and institutional domains.

This study, therefore, aims to explore how RDPAGs in Poland engage with educational gaps in the RD landscape. Specifically, it examines the strategies, challenges, and perceived impact of patient-led educational efforts directed toward families, healthcare professionals, and the general public. A combination of these issues led to the following research questions: (1) how do RDPAGs support families with RD in terms of information, education, and peer guidance? (2) What strategies do they use to raise awareness and improve knowledge about RDs among professionals and the public? (3) What challenges and opportunities do they face in fulfilling this educational role within the Polish healthcare context?

## 2 Methods

### 2.1 Study design

This study forms part of a broader project examining the role of patient-led organizations in the RD landscape, with the present analysis focusing specifically on their educational and informational functions. Since no prior research has explored this topic in Poland, the study employed a Qualitative Descriptive (QD) design (47), which is particularly suitable for exploring under-researched, practice-oriented topics using participants’ natural language. The study was designed to examine leadership-based perspectives on educational and advocacy practices within RDPAGs, rather than to provide comprehensive or representative accounts of the entire RD community. In this design, organizational leaders were treated as key informants because they are responsible for shaping educational strategies, engaging external stakeholders, and negotiating organizational roles within healthcare, educational, and policy contexts.

The QD approach aims to provide rich, straightforward descriptions of phenomena without imposing theoretical abstraction or pre-existing frameworks. It allowed the exploration of how RDPAGs in Poland engage in educational and informational activities across multiple sectors. The study was based on individual in-depth interviews with 11 leaders of RDPAGs, including foundations and associations supporting individuals with rare conditions and their families.

This study adhered to the Consolidated Criteria for Reporting Qualitative Research (COREQ) (48). The COREQ framework was used to structure the reporting of study design, data collection, reflexivity, analysis, and findings. While COREQ has been critiqued for its partial misalignment with reflexive thematic analysis (49), it remains a widely used tool for ensuring clarity and transparency in qualitative health research reporting. Thus, the checklist was applied pragmatically to support a comprehensive and transparent presentation rather than as a strict methodological guide.

The study was guided by three research questions addressing the educational strategies, audiences, and systemic challenges faced by RDPAGs in Poland. By highlighting the voices of organizations involved in education and advocacy, the study offers insight into how RDPAGs operate within the Polish RD ecosystem and their implications for policy, professional training, and future support initiatives.

### 2.2 Participants and setting

Eligibility criteria included: age 18 or older, active involvement in an RD organization for at least 1 year, holding a leadership role, willingness and ability to participate in an online interview, informed consent, fluency in Polish, and access to the Internet.

A purposive sampling strategy was used. Organizational leaders were selected as participants because they are directly involved in designing, coordinating, and representing educational and advocacy activities at the interface between patient communities and healthcare, educational, and policy institutions. This positioning makes them particularly well-suited to reflect on organizational strategies, target audiences, and system-facing challenges relevant to the research questions.

Participants were recruited through the Facebook pages of relevant patient associations and foundations working in the RD field, with the invitation explicitly addressed to individuals holding leadership or coordinating roles within these organizations. This open call described the study’s purpose and eligibility criteria, and interested individuals contacted the researcher directly.

Of the 12 individuals who initially responded to the call, 11 ultimately participated. One person declined due to time constraints, and another withdrew before the interview but referred a colleague from the same organization, who joined the study instead.

All participants were leaders of RDPAGs and primary caregivers to individuals with RDs, reflecting the empirical organization of the RD advocacy field, in which patient organizations are typically founded and led by parents or patients. While this dual positionality was not an *a priori* sampling criterion, it shaped how participants discussed the translation of lived experience into organizational educational and advocacy practices.

### 2.3 Research tool

The interview schedule was developed specifically for this broader research project using the Qualitative Pretest Interview method (51), drawing on the literature on informal caregiving, peer support, and patient-led advocacy in RD communities.

Although the full schedule was designed to broadly explore how caregivers build, maintain, and benefit from support networks, the present study focuses only on a subset of questions addressing the educational and informational functions of RDPAGs. These included, for example, the types of informational support provided to community members and broader educational activities aimed at raising public awareness of RDs (Supplementary material).

Two pilot interviews were conducted to refine the instrument, identify potential biases, and improve research logistics, leading to minor revisions and enhancing both methodological and social validity.

The final schedule was reviewed by two patient-organization board members and one medical sociologist and was approved by the PUMS Bioethics Committee.

### 2.4 Data collection

Due to the geographic dispersion of RD communities and limited participant availability, interviews were conducted via MS Teams in July 2025. Ten interviews were audio-video recorded, while one participant, due to scheduling constraints, provided written responses to the same interview guide. The written response followed the same semi-structured interview guide and addressed all core analytic domains. It was treated as supplementary material and used primarily to support and contextualize themes identified across the interview dataset, rather than as a primary driver of theme development. No themes were generated solely on the basis of the written account. The written account was reviewed for completeness and thematic relevance and included in the final dataset.

In total, 11 interviews were completed. All interviews were conducted by the author, a medical sociologist with experience in RD research and qualitative methodologies. Each interview lasted approximately 70 min, yielding over 12 h of audio material. Most participants joined from home, fostering a sense of comfort and privacy.

Transcriptions were prepared using the built-in Microsoft Teams transcription tool and were manually verified for accuracy against the audiovisual recordings. While participants did not request transcript validation, they were encouraged to clarify or expand their statements during the interviews if needed. Follow-up interviews were deemed unnecessary due to the richness and clarity of the collected data.

Recruitment and analysis proceeded iteratively until the data provided sufficient depth and richness to support coherent and well-developed themes (52, 53). By the ninth interview, the overall thematic structure was conceptually clear, and subsequent interviews served to refine and deepen existing themes rather than generate new ones.

### 2.5 Data analysis

Interviews were conducted with representatives of organizations supporting individuals with various rare and ultra-rare conditions, including neurological, genetic, and metabolic disorders. To ensure anonymity, all identifying details, such as specific disease names or organizational affiliations, were removed from the transcripts and the analysis.

The data were analysed using Reflexive Thematic Analysis (RTA) following Braun and Clarke’s six-phase framework, which provides a flexible yet rigorous approach for identifying, developing, and interpreting patterns of meaning in qualitative data (54, 55). The analysis was semantic and inductive, meaning that codes and themes were constructed from participants’ surface-level meanings rather than imposed through a theoretical lens (54). The process was grounded in a constructivist-interpretivist paradigm (55–57).

After familiarization with the transcripts, the researcher generated open, descriptive codes by systematically reviewing participants’ explicit responses. These initial codes were clustered into candidate themes based on conceptual relatedness. Themes were then reviewed for internal coherence, refined recursively, and clearly defined through further engagement with the data. Final themes were named, described, and supported with illustrative participant excerpts.

This method aligned with the study’s Qualitative Descriptive design, which prioritizes low-inference interpretation, close attention to participants’ language, and practice-relevant findings. The use of semantic and inductive RTA is congruent with QD methodology, as both emphasize staying close to participants’ accounts and avoiding abstract theorizing (56). All coding and theme development were carried out manually, without the use of qualitative data analysis software.

Data collection and analysis proceeded iteratively. By the ninth interview, key themes were conceptually well developed and richly supported; the final two interviews helped reinforce, refine, and elaborate the theme structure. While the concept of “saturation” has been debated in RTA (52), the researcher judged that the dataset had reached sufficient information power to support the analytic claims being made.

To enhance analytic rigour, an audit trail was maintained, documenting decisions about coding and theme development. Reflexivity was practiced throughout the study through memo-writing and critical self-reflection, acknowledging how the researcher’s disciplinary background in medical sociology and prior experience in RD research influenced the analytic process. This positionality shaped analytic sensitivity to issues of advocacy, power, and knowledge asymmetries within health and social systems, and was treated as an analytic resource rather than a source of bias (53, 55).

Although coding and theme construction were carried out by a single researcher, analytic rigor was ensured through prolonged engagement with the data, iterative theme refinement, reflexive memo-writing, and the maintenance of a transparent audit trail documenting analytic decisions. Consistent with RTA, rigor was conceptualized in terms of reflexivity, coherence, and transparency rather than inter-coder agreement (57).

The use of RTA was intentionally aligned with the QD design of the study. While QD prioritizes low-inference, practice-oriented accounts grounded in participants’ everyday language, RTA offers a flexible analytic framework that enables systematic pattern identification without imposing pre-existing theoretical constructs. In this study, RTA was applied in a predominantly semantic and inductive manner, consistent with the aims of qualitative description, allowing themes to remain close to participants’ accounts while supporting reflexive engagement with meaning-making processes, rather than theorizing beyond the data.

## 3 Results

### 3.1 Study participants

The study included 11 representatives of RD patient organizations (Table 1). Participants ranged in age from 36 to 53 years (mean age: 41.8 years) and included nine women and two men. All held leadership positions within their organizations, with six serving as presidents, two as vice presidents, and three as board members.

**Table 1:** Study participants.

Characteristic	Details
Age (years)	Range: 36–53 Mean: 41.8
Gender	Female: 9 Male: 2
Position in the organization	President: 6 Vice-president: 2 Board member: 3
Years of involvement in the organization	Range: 1–9 Mean: 4.7
Size of organization (members)	Range: 50–800 Mean: 291 < 100: 5 101–200: 2 201–500: 2 501–1,000: 2
Scope of activity	National: 3 National/International: 8
Relationship with a person with RD	Parent: 10 Patient: 1
Worldwide prevalence of the disease	1:2000–1:50000: 9 <1:1000000: 2

Their involvement in the organization varied from 1 to 9 years, with a mean of 4.7 years. The size of the organizations they represented ranged from 50 to 800 members, with an average of approximately 291 members. In terms of operational scope, two organizations operated at the national level, while the remaining seven combined national and international activities. Regarding personal connection to RD, 10 participants were parents of affected individuals, and one was a patient themselves. Nine participants were linked to diseases affecting between 1 in 2,000 and 1 in 50,000 individuals globally, while two were associated with ultra-rare conditions diagnosed in fewer than 1 in 1,000,000 people.

### 3.2 Themes and subthemes

The analysis identified a central theme capturing how participants described RDPAGs as acting as intermediaries of knowledge, often referred to by leaders as “ambassadors.” This role was articulated in relation to translating experiential knowledge into educational, advocacy, and system-facing practices (Table 2). Within this overarching theme, three interrelated subthemes were identified: (1.1) acting as trusted and up-to-date knowledge holders, (1.2) working to bridge knowledge gaps across different stakeholder groups, and (1.3) engaging in advocacy aimed at increasing systemic awareness and recognition of RDs.

**Table 2:** Themes and subthemes emerging from qualitative analysis.

Theme	Subthemes	Interpretive description
1. Ambassadors of Knowledge	1.1. Trusted and up-to-date knowledge holders	RDPAGs serve as reliable sources of current, verified medical and experiential knowledge for families, professionals, and institutions.
	1.2. Bridging knowledge gaps across stakeholders	RDPAGs work to reduce knowledge asymmetries between patients, caregivers, professionals, and policymakers.
	1.3. Advocacy for systemic awareness and recognition of RDs	RDPAGs actively promote recognition of rare diseases at the systemic level and advocate for their inclusion in health and education policies.
2. Navigating Uncertainty: Supporting Families Through Experiential Knowledge	2.1. Early-stage informational support after diagnosis	RDPAGs provide crucial guidance and clarity to families following diagnosis, helping to reduce confusion and isolation.
	2.2. Ongoing education and access to curated, reliable knowledge	RDPAGs offer continuous, tailored educational resources through webinars, guides, and direct support.
	2.3. Peer-to-peer mentoring	RDPAGs facilitate emotional support and experiential knowledge exchange among caregivers with shared challenges.
	2.4. Participation, responsibility, and community engagement	RDPAGs empower families to become active participants in advocacy and community-building, fostering confidence and collective agency.
3. Challenging Expertise: Shaping Professional Practice from the Margins	3.1. Educating healthcare professionals	RDPAGs educate healthcare providers to improve understanding and management of rare diseases.
	3.2. Teaching education sector professionals	RDPAGs support school staff in addressing the learning needs of children with rare conditions.
	3.3. Joint training and co-creation of guidelines/resources	RDPAGs collaborate with professionals to co-create practical tools and inclusive standards of care.
4. Claiming Visibility: Advocacy as Public Education and Systemic Engagement	4.1. Public awareness campaigns and media outreach	RDPAGs organize public campaigns to raise awareness and reduce stigma around rare diseases.
	4.2 Peer-to-peer education between organizations	RDPAGs share resources and strategies with other advocacy organizations to strengthen collective capacity.
	4.3. Institutional education and policy advocacy	RDPAGs participate in policy consultations to influence systemic and legislative reforms in healthcare and education.

Building on this core theme, the analysis identified three broader domains that reflect the main contexts in which these practices were enacted, as described by participants: (1) supporting and educating patients and families, (2) engaging with professionals and contributing to professional learning, and (3) raising public visibility and participating in policy-oriented advocacy. Together, these themes capture how participants narrated their organizations’ educational and advocacy activities across medical, social, and institutional settings.

#### Theme 1: ambassadors of knowledge

Interviewees positioned RDPAGs as knowledge brokers and described an educational mandate focused on providing accurate and up-to-date information to families, healthcare professionals, educators, and the broader public. They emphasized that this role draws on both scientific evidence and lived experience, which leaders framed as enabling reliable, practical guidance. Participants also described advocacy-oriented activities, such as ongoing dialogue with medical professionals and decision-makers, aimed at improving recognition of RDs and addressing the needs of affected families. Across accounts, leaders reported efforts to counter misinformation, raise awareness, and engage institutions across sectors through sustained outreach and collaboration.

##### 1.1 Trusted and up-to-date knowledge holders

Although social support was often perceived as the most visible function of RDPAGs, leaders argued that their educational work is equally central. They emphasized that access to accurate knowledge is not only complementary to psychosocial support but, in their view, a prerequisite for effective care and longer-term empowerment of families.

*We have prepared a knowledge base divided into three sections: therapists, doctors, and parents, where there are materials dedicated to specific groups.* (S6)

*The educational role is somewhat downplayed or pushed aside, with everyone talking about the need for social support, but I think that, paradoxically, this educational role is very important.* (S8)

Leaders also described sustained efforts to bridge awareness gaps across stakeholder groups, including healthcare professionals, educators, and the broader public. Their accounts referred to cooperation with doctors, conference organization, and attempts to strengthen recognition within the medical community, alongside addressing misunderstandings encountered in schools and other institutions.

*We inform patients, but we also cooperate with doctors and organise conferences. We are active on the Internet. (…) We are recognised and known in the medical community.* (S5)

*The biggest challenge for families is the lack of awareness about this syndrome among all the people who have to deal with a child with* [name of syndrome] *in a professional community, whether they are doctors, psychologists, or teachers. It is a challenge because they do not know about this syndrome, and they are not always willing to learn about it. Some do, but some lump our children into one category, and we have to fight against that (…) at every stage, at the doctor’s, at school, everywhere.* (S6)

##### 1.2 Bridging knowledge gaps across stakeholders

Participants described a range of practices through which RDPAGs seek to make RD-related knowledge more visible and accessible across communities and institutions. Their accounts emphasized sustained dissemination efforts and public presence, which leaders framed as gradual attempts to influence perceptions and increase recognition among healthcare professionals, educators, and the broader public.

*We constantly promote this knowledge so that it can be put into practice somewhere, and we can see that we are becoming increasingly visible (…) in various institutions. Slowly, slowly, like a drop hollows a stone.* (S10)

Several participants highlighted how prolonged caregiving experience was perceived as leading to the accumulation of condition-specific expertise. Leaders described this process as driven by necessity, particularly in contexts where formal guidance was limited or unavailable.

*It is often us, the parents and caregivers, who, after many years of caring for our children, become somewhat of experts and specialists when it comes to therapy. For example, even though I am a* [profession]*, I could certainly talk a lot about* var*ious forms of physiotherapy. (…) we, the caregivers, have no choice; we have to be knowledgeable, and we have to explore the subject, often searching for information on the internet.* (S7)

Beyond individual expertise, participants described the collective, experience-based knowledge accumulated within RDPAGs as increasingly recognized in interactions with professionals. In their narratives, this recognition was framed as a shift toward more dialogical relationships with medical experts and decision-makers, in which experiential knowledge was positioned alongside scientific evidence.

*Perhaps it is because we have this practical knowledge, and in the case of rare diseases, this knowledge is just as valuable as the theoretical knowledge we can find in scientific papers. So this attitude has certainly changed; we are treated as partners.* (S2)

##### 1.3 Advocacy for systemic awareness and recognition of RDs

Participants portrayed RDPAGs as occupying intermediary positions between families, clinicians, and policymakers. They described advocacy activities aimed at translating scientific knowledge into practical guidance for healthcare providers and patients, with the stated goal of improving recognition and understanding of RDs within the medical system.

*As a patient organisation, we talk to doctors and therapists about what is happening. We are ambassadors, we are advocates, and in fact, I believe that the role of patient organisations as a link between patients and doctors is extremely important. We have certain opportunities to transfer this knowledge from scientists to doctors and families.* (S8)

The metaphor of “ambassadors,” introduced by participants, reflects how some leaders conceptualized their role as mediators between professional and lay domains. At the same time, this metaphor implicitly positions RDPAGs as actors endowed with legitimacy, authority, and recognition, thereby rendering less visible the tensions, limits, or contestations that may accompany such intermediary roles. In these accounts, the knowledge shared was described as both scientific and experiential, shaped by caregiving trajectories, advocacy work, and collaboration with researchers and clinicians.

Participants also reported extending their advocacy beyond professional settings into the broader public sphere. These activities were framed as efforts to raise societal awareness and promote greater openness toward the needs of people living with RDs and their families.

*As with many rare diseases, caregivers often become medical experts, organisers of everyday life, rehabilitation specialists, and advocates for their children (…) We also want to build greater awareness and openness in society to the needs of people with* [name of syndrome] *and their families.* (S9)

##### Theme 2: navigating uncertainty: supporting families through experiential knowledge

Across interviews, participants described a range of educational and supportive practices directed at families affected by RDs. These practices were framed as responses to uncertainty surrounding diagnosis, care pathways, and everyday caregiving. Leaders spoke about providing factual guidance alongside emotional reassurance, with activities spanning different stages of the illness trajectory, from early informational support following diagnosis to ongoing access to resources, peer mentoring, and community-based interactions. In participants’ accounts, these initiatives were described as shaping how families navigate the practical and emotional challenges of RD caregiving.

###### 2.1 Early-stage informational support after diagnosis

When discussing educational activities aimed at parents, participants consistently emphasized the period immediately following diagnosis as particularly challenging. Early-stage informational support was described as focusing on practical and actionable guidance, including lists of recommended specialists, diagnostic pathways, and advice on where to seek further assistance. Leaders framed these efforts as attempts to help families orient themselves within unfamiliar and often overwhelming healthcare systems.

*First of all, we focus on specific actions because sometimes families of newly diagnosed children come to me, and they are completely unaware of what awaits them. We have prepared guides on which specialists to consult and what detailed diagnostics to perform.* (S2)

*Giving others advice about what I would have liked to know when I was starting, and I think there is nothing more valuable at the beginning.* (S4)

Alongside practical information, participants highlighted the personal and experiential dimension of early support. Experienced caregivers described sharing their own stories and reflections as a way of offering perspective and reassurance to newly diagnosed families. These exchanges were portrayed as addressing not only informational needs but also the emotional impact of diagnosis.

*I speak primarily as a mother, because I remember myself from that time (…) We receive feedback that it helped them understand, look at certain things differently, gain perspective, gain hope.* (S8)

*We developed (…) a compact guide for families with a newly diagnosed child, containing all the most essential information to help them understand and navigate their situation (…) It also includes the phone numbers of association members who have agreed to have their numbers published. And when a new family joins and needs more information, they can contact us directly and talk to us.* (S7)

###### 2.2 Ongoing education and access to curated, reliable knowledge

Participants described maintaining curated repositories of information, most commonly in the form of organizational websites or online platforms. These repositories were said to include step-by-step diagnostic and care guidance, lists of recommended specialists and treatment centres, and summaries of current medical knowledge. In participants’ accounts, such resources were developed in response to families’ frequent reliance on fragmented or unverified online information.

*We run a website that is the largest knowledge base about* [name] *syndrome in Poland (…) we have a large information guide about the disease and genetic testing. We also have a whole subpage with recommended centres and doctors recommended by our association (…) The entire diagnostic process is described*. (S5)

Participants framed these materials as tools intended to support families in orienting themselves within complex care systems and making decisions aligned with their individual circumstances, including geographic and financial constraints.

*Parents often ask on various internet forums where to take their child, what therapies are available, and which doctors are familiar with* [name] *syndrome. If we create such a guide, parents simply get a ready-made product and check, for example, where the closest place is, where they can go due to their financial situation or for the therapy they care about (…). We organise meetings with a psychologist and a sexologist about our children’s puberty, and a meeting about incapacitation.* (S7)

Beyond static materials, participants reported organizing ongoing educational activities such as webinars, conferences, and specialist-led meetings. These initiatives were described as addressing medical developments (e.g., clinical trials), legal and psychosocial issues, and practical life skills, and were often tailored to different audiences, including parents, healthcare professionals, and therapists. Some participants emphasized the importance of continuity and accessibility of these educational resources, noting that recorded materials enabled ongoing access for families who joined the community at later stages.

*We educate and inform* [about clinical trials]. *We also held meetings online (…), and more people understand what clinical trials are (…) That is why we organise these conferences (…) we talk about what scientists and doctors say, and not what people who collect money for sick children say, claiming that this medicine will cure or slow down the disease, because that is not true.* (S3)

*These are webinars about the challenges faced by the syndrome (…). They are available in this group, so when you join the group, you have access to a fairly large database of information from previous recordings. (…) In addition, there are meetings, webinars, not on the subject of the syndrome itself, but on how to deal with this situation.* (S6)

###### 2.3 Peer-to-peer mentoring

Peer-to-peer mentoring was repeatedly described as a central educational practice within RDPAGs. Participants portrayed mentoring as facilitating the exchange of experience-based knowledge between families, particularly by connecting newly diagnosed parents with more experienced caregivers. These interactions were framed as spaces for sharing practical strategies, navigating healthcare pathways, and discussing everyday challenges of caregiving.

*When a new family comes to me right after a diagnosis (…), the very possibility of sharing one’s experiences and supporting someone is a big thing (…), namely, contacts, exchange of experiences, and above all, the feeling that one is understood, because the other parent has either gone through a similar situation or the same one. (…) We know from our own experience.* (S2)

Participants associated trust within mentoring relationships with shared caregiving experience, including familiarity with specific treatments, dietary practices, and interactions with specialists. Drawing on others’ prior experiences was described as a way of avoiding challenges previously encountered by peers.

*The baggage and experience we have as parents is enormous (…) parents often follow the path set by other parents (…). Why should I make mistakes that have already been made before, when I can draw on the experience of another parent and avoid making the mistakes that they once made?* (S7)

Peer interactions occurred in various formats, including one-to-one conversations, online meetings, and closed-group webinars. Participants described these spaces as combining informational exchange with emotional support, extending beyond immediate practical issues to include coping strategies related to diagnosis and long-term caregiving. Some leaders also reported providing ongoing, case-specific advice based on accumulated experiential knowledge.

*The most effective thing is to share your own stories, mutual empathy, and strengthening hope (…) The main problems parents face are that they lack knowledge, they don’t know where to go, which specialist to see (…) even this form of support, such as talking to a parent and directing them to a particular institution, for example, is already a solution to the problem of providing help.* (S9)

*I consult with other cases of* [name] *syndrome on an ongoing basis, providing advice or administration. Whether correct, dietary, medicinal, i.e., how to introduce, how to wean off.* (S4)

###### 2.4 Participation, responsibility, and community engagement

Participants described community engagement within RDPAGs as involving negotiated expectations around participation and responsibility. Leaders noted tensions between informal participation in online support spaces and formal organizational membership, particularly in relation to sustaining educational and advocacy activities.

*Recently, we have been informing them that this is not a support group and that these webinars do not come out of nowhere, that someone is doing this, is devoting their time (…) They did not understand the difference between being a formal member (…) and being in a support group on Facebook (…) there were 150 people at the convention, there are 681 people in the support group at the moment (…) and there were 40 people in the association.* (S6)

These accounts point to internal tensions between expectations of solidarity and the practical demands of sustaining organizational work, suggesting that empowerment within RDPAGs may also involve negotiation, boundary-setting, and moments of frustration. In this context, leaders framed increased involvement, including financial contributions, as necessary for maintaining and expanding organizational activities. These accounts highlight how educational and advocacy work is intertwined with organizational sustainability.

Participants also described deliberate efforts to reframe collective experiences of living with an RD. Leaders spoke about emphasizing positive narratives during meetings, webinars, and campaigns, presenting community engagement as a space for connection and mutual recognition alongside ongoing challenges.

*We started to strongly emphasise that when we meet, we do it with positive energy (…) to show the enormous value of the fact that we are here, that we would never have met if our children had not been diagnosed, (…) reversing this perspective from the burden of the disease to the positive that can be found in it (…) We show that behind this grey hole one is in now, there is a great organisation, great people who live their lives, cope with this situation, and there is a light.* (S6)

Rather than eliminating hardship, these narratives were described as shaping how members interpret and share their experiences within the community.

##### Theme 3: challenging expertise: shaping professional practice from the margins

Participants consistently framed professional knowledge deficits as a central obstacle to effective care for individuals with RDs across healthcare, education, and related sectors. In their accounts, limited awareness among professionals was associated with delayed diagnoses, constrained access to appropriate interventions, and increased burden on families. Against this background, participants described RDPAGs as assuming educational and mediating roles aimed at addressing perceived gaps in professional knowledge through information provision, dialogue, and collaborative initiatives. These activities were presented as efforts to improve recognition, referral practices, and care coordination across health and education settings.

###### 3.1 Educating healthcare professionals

Participants frequently described encounters with healthcare professionals whom they perceived as lacking familiarity with specific RDs, particularly at the level of primary care and general specialization. According to participants, this limited awareness contributed to delayed referrals, missed opportunities for genetic testing, and frustration for families navigating the diagnostic process. At the same time, participants acknowledged the structural constraints of medical practice, noting that it is unrealistic for individual clinicians to possess detailed knowledge of all rare conditions.

*The specialists we encountered were random, local ones. They still didn’t know what we were talking about (…) So it was clear to us that the medical community in general was unfamiliar with this syndrome. This was true of both general practitioners and specialists, including psychiatrists.* (S6)

*Challenges resulting from the ignorance of doctors who (…) who do not see the need to order genetic testing (…). We hear doctors saying, “You don’t look like you need it. Why do these tests?”.* (S5)

At the same time, participants distinguished between expectations of comprehensive expertise and what they framed as a minimal threshold of recognition and referral competence.

*It is clear that GPs and paediatricians cannot be expected to have knowledge about 10,000 rare diseases, but they should at least know that such a syndrome exists and be able to refer patients for genetic testing.* (S7)

In response to these perceived gaps, participants described RDPAGs as engaging in targeted educational activities aimed at healthcare professionals. These included producing brochures, leaflets, calendars, and printed guides intended to raise awareness of specific conditions and support basic diagnostic and referral processes.

*It is necessary to educate people about the existence of this disease, how to diagnose it, e.g., by sending leaflets and calendars to all public and private medical practices.* (S4)

*These are also materials that we provide to specialists, doctors, and therapists. (…) So this is one of the methods, i.e., publishing.* (S2)

Participants also described initiating direct dialogue with clinicians, drawing on their caregiving experience to supplement biomedical knowledge. While some professionals were described as resistant to parental input, others, particularly physiotherapists and allied health professionals, were portrayed as more open to collaboration. These mixed reactions illustrate that the positioning of RDPAGs as knowledge partners is not uncontested and may depend on professional hierarchies, disciplinary cultures, and perceived boundaries of expertise.

*Some specialists think that since you are a parent, you have come to a specialist, let them do their job, and they don’t need* [your advice] *(…). But in the case of physiotherapists (…) they are happy when we come to them with a new idea (…), for example, the therapists said, “Great, we didn’t know about such a thing at all, it’s cool that you showed it to us. Is there any possibility of training?” So I organised training for them with a representative of this company.* (S7)

In these accounts, RDPAGs were framed as intermediaries who facilitate communication between families and clinicians by accompanying diagnoses with informational materials and contact details, thereby linking medical care with psychosocial and practical support.

*We created leaflets that we sent to genetic counselling centres and paediatric neurology clinics, so when a person receives a diagnosis, they also receive a leaflet with our contact details (…). I think it also makes it easier for doctors, because we are simply a link, and I think that is just so important.* (S8)

###### 3.2 Teaching education sector professionals

Participants similarly emphasized limited awareness and preparedness among education professionals. Even when diagnoses were formally established, teachers were described as lacking condition-specific knowledge and practical guidance on how to support children with RDs in classroom settings. While participants described that there is a widespread lack of preparation for recognizing and responding to the specific educational needs of children with RDs, they also suggested that children without visible symptoms were often perceived as not needing support, which they associated with inadequate classroom accommodations.

*When a child goes to school (…) No one knows what this syndrome is. Even those teachers who already know that the child has been diagnosed (…) and want to help, they don’t know how. I have heard many times, “I would even like to know how to work with* [child’s name]*, but I don’t know how, because no one has prepared me for this. I’m just a regular maths teacher. I have no idea what his educational needs are”. So our activities then concerned not only doctors, but also the educational environment (…)* [we educate them on] *how to work with these children.* (S6)

Participants associated these gaps with challenges in securing appropriate educational accommodations, particularly for children whose conditions were not visibly apparent.

*Our patients, if they are well cared for from childhood, do not have such strong, visible physical characteristics of* [name] *syndrome and problems, and they also hear in schools (…) that the child does not look like they need to be given so many concessions, that it is not necessary.* (S5)

In response, participants described RDPAGs as initiating educational activities aimed at schools and educational institutions. These included publishing guides on inclusive education, collaborating with local authorities, and participating in pedagogical experiments. These initiatives were described as aiming at raising awareness and building competencies in working with children affected by RDs within school settings.

*We have published a book on inclusive education. We are conducting a pedagogical experiment with the city.* (S1)

*We publish guides (…) which can be taken to schools and various institutions.* (S10)

Participants also reported engaging with teachers, students, and academic institutions through conferences and research collaborations. While these interactions were described as increasing educators’ interest in RDs and positioning caregivers’ experiential knowledge as a resource for professional learning.

*When students are looking for topics for their research papers (…) As an association, we offer them help, i.e., we pass on this practical knowledge, theoretical knowledge, materials (…), our stories are very valuable data (…), our research results or our stories can be of value to scientists and specialists.* (S2)

The reported scale of some initiatives was framed as evidence of unmet demand for knowledge among educators.

*We managed to organise a nationwide conference for educators and teachers (…) We had 300 people in the room, and we only talked about the syndrome, what these children are like, what the symptoms are, how to work with them (…) the interest was enormous.* (S6)

*We are preparing a database on the assessment of schools’ preparedness for people with disabilities. In terms of, let’s say, construction and care, here we want to help children with disabilities at school (…) We want to make a breakthrough.* (S3)

###### 3.3 Joint training and co-creation of guidelines/resources

Participants described involvement in joint training initiatives and the co-creation of practical resources, including clinical guidelines and coordinated care models. These activities were framed as combining international standards with experience-based knowledge derived from caregiving and advocacy work.

*Work is underway on clinical guidelines for* [name] *syndrome (…) covering every field, both medical and non-medical, such as cardiology, neurology, sexology, psychology, child development (…) for both parents and specialists.* (S2)

*We will develop such a* [coordinated care model project] *based on international guidelines for the care of* [name of disease]. (S3)

Beyond healthcare-specific initiatives, some participants reported engagement in broader policy-oriented projects related to accessibility and disability inclusion. These included developing minimum standards for municipalities and proposing systemic solutions aimed at improving participation and access for people with RDs.

*We have developed minimum standards within the metropolis (…). to improve accessibility for people with special needs (…). We have also created and proposed what is now referred to as the ‘disability package’ for the city card.* (S1)

Across these accounts, participants portrayed RDPAGs as operating at the intersection of experiential knowledge, professional practice, and institutional frameworks, seeking to influence how RDs are recognized and addressed within established systems.

##### Theme 4: claiming visibility: advocacy as public education and systemic engagement

Beyond activities directed toward families and professionals in healthcare and education, participants described RDPAGs as engaging in a range of practices aimed at increasing the public visibility of RDs and strengthening their recognition within institutional settings. These practices were framed as forms of public education and systemic engagement, encompassing media campaigns, policy-oriented advocacy, and inter-organizational knowledge exchange. Participants emphasized the strategic use of personal caregiving experience alongside planned communication when engaging decision-makers across multiple levels.

###### 4.1 Public awareness campaigns and media outreach

Participants described public awareness campaigns as a key mechanism through which RDPAGs seek to make RDs visible in the public sphere. These initiatives ranged from symbolic actions, such as illuminating public buildings in specific colours, to coordinated nationwide campaigns covered by traditional and online media. According to participants, such activities were intended to stimulate public recognition, foster solidarity, and create opportunities for broader discussion.

*As an association, we illuminate buildings in* [colour] *wherever possible. Everywhere, everywhere (…). parents have been coming together on that day to take photos in front of these buildings (…) and socialise locally. Or in kindergartens, children dress up (…) to raise awareness.* (S6)

*We got involved in the* [name of the campaign] *campaign during which we lit up Poland in* [colour] *because it is an amazing sign of unity and support for people with the syndrome.* (S7)

*I organised a nationwide educational campaign* [name]*, which was widely reported in the media (…) its aim was to spread information about* [name] *syndrome and the challenges faced by families.* (S4)

Participants also described extending outreach beyond symbolic campaigns by engaging academic, cultural, and artistic platforms. These activities included participation in scientific conferences, organizing popular science events, hosting concerts, and co-creating exhibitions. In participants’ accounts, such initiatives were intended to reach audiences not typically engaged through health-focused advocacy alone.

*We participate in scientific conferences at universities and other organisations (…) where we have already spoken several times about the syndrome and the problems faced by people with rare diseases. We also participate in the activities of the* [name] *social movement, which brings together associations and foundations for rare diseases.* (S2)

*We are organising a popular science conference (…) with over 50 lectures (…) by people who talk about various aspects of the lives of people with rare diseases. (…) we have been organising Rare Disease Day, and a concert* [name] *to raise awareness through music (…), an exhibition (…) presented by various artists.* (S1)

These accounts position public visibility not only as an outcome but as an ongoing process requiring repeated symbolic, cultural, and educational interventions. However, sustaining such high-visibility activities was occasionally described as resource-intensive and emotionally demanding, particularly for a small number of highly engaged leaders. At the same time, the emphasis on visibility and positivity may obscure less publicly shareable experiences of fatigue, conflict, or marginalization, which were largely absent from leaders’ narratives.

###### 4.2 Peer-to-peer education between organizations

Participants described collaboration with other patient organizations as an important dimension of advocacy and knowledge exchange. Participation in national and international networks was framed as enabling the circulation of strategies, resources, and organizational practices, while also supporting collective visibility within the broader RD field. Some leaders also noted that their organizations aim to inspire other groups to adopt similar approaches and expand their activities.

*We have joined the Council of Patient Organizations at the Patient Rights Ombudsman and the Federation of Patients at the National Orphan Forum. We are also in constant contact with the other* f*oundations. So we cooperate and draw on the experience of these other foundations.* (S7)

*I feel that by being there, undertaking these activities, promoting the mission, goals, and overall activities of EURORDIS, we are somehow encouraging other organisations to do the same. And the same goes for ERN* [European Reference Network]*, right?* (S1)

These collaborations were described as facilitating mutual learning and informal mentorship between organizations with differing levels of experience, capacity, and resources. Participants suggested that showcasing organizational activities to peers served both practical and symbolic purposes, reinforcing a sense of shared movement while encouraging newer groups to expand their activities.

*We draw on the experience of other patient organisations, which enables us to operate effectively.* (S10)

*Whatever we do (…) we show them that this is not the last piece of the puzzle. It is one of the most important pieces.* (S6)

Such accounts emphasize inter-organizational learning as both a pragmatic strategy and a form of collective identity-building within the RD advocacy landscape.

###### 4.3 Institutional education and policy advocacy

Participants framed institutional education and policy advocacy as responses to persistent gaps in how RDs are recognized and addressed within public systems. Leaders described recurring challenges in disability assessment procedures, where individuals with specific syndromes were perceived as insufficiently impaired to qualify for support.

*They look “too healthy” for a certain degree of disability, or we hear comments like “Let’s give them a mild one”.* (S5)

*At the systemic level, another difficulty is the lack of sufficient knowledge about* [name] *syndrome (…) among public institutions. This causes delays in diagnosis, difficulties in obtaining appropriate educational or care support, and a lack of access to specialist services that should be standard.* (S9)

In response, participants described engaging in activities aimed at educating public officials and influencing policy processes. These included participation in parliamentary committees, public consultations, advisory bodies, and working groups addressing disability and healthcare legislation. Advocacy efforts were presented as combining lived caregiving experience with formal, system-oriented engagement.

*We participated in the development of minimum standards (…). We co-created or advised on the development of the province's strategy.* (S1)

*We participate in work on disability legislation and changes in legal regulations.* (S2)

Participants also suggested that formal organizational status enables patient associations to contribute to regulatory and reimbursement processes, such as issuing opinions on specific therapies or participating in deliberations within national health technology assessment frameworks.

*Our association participates in the meetings of the Parliamentary Group on Rare Diseases (…), mainly intended to reach the relevant ministries, and highlight the needs and barriers faced by patients and caregivers, or fight for improved access to modern medication.* (S4)

Across these accounts, advocacy was framed as a form of public and institutional education through which RDPAGs seek to influence how RDs are understood, categorized, and addressed within policy and service delivery systems.

## 4 Discussion

In the absence of accessible information and widespread medical knowledge about rare conditions, this study examined how RDPAGs are positioned, and position themselves, as knowledge brokers, educators, and policy actors within structurally constrained healthcare and education systems. By combining scientific evidence with experience-based insight, participants described how these organizations attempt to bridge critical information gaps for families, healthcare providers, educators, and public institutions. They not only educate caregivers about diagnosis, symptom management, and care coordination but also actively engage in educating healthcare and education professionals, developing joint training initiatives, and co-creating guidelines that integrate clinical standards with practical, family-informed knowledge. Moreover, they extend their influence into the public and institutional spheres through awareness campaigns, inter-organizational collaboration, and policy advocacy aimed at systemic change. The central theme of RDPAGs as “ambassadors of knowledge,” developed from participants’ accounts, captures how these multiple roles are narrated and legitimized by organizations’ representatives. It underpins and connects the three domains in which this role is enacted: education and empowerment of families, collaboration with professionals, and broader public and policy advocacy.

Firstly, participants framed RDPAGs as intermediaries who seek to bridge scientific evidence with experience-based insight, positioning their work as extending beyond the provision of accurate information to families. In their accounts, RDPAGs also educate professionals and institutions, counter misinformation, and advocate for greater systemic recognition of RDs. Through sustained outreach and visibility, leaders described attempts to enhance awareness and responsiveness within healthcare, education, and public services, although the success of these efforts varied across institutional contexts. These findings resonate with a substantial body of international literature describing RD patient organizations as hybrid actors situated between lay experience, biomedical expertise, and institutional governance (39, 40, 58). Previous studies have shown that RDPAGs function as emotional and practical hubs for newly diagnosed families, guiding them through care pathways, connecting them with specialists, and offering peer support (39, 40, 58–60), while also contributing to biomedical research through registries, trial recruitment, and agenda-setting (60–63). This perspective supports holistic, person-centred approaches to system change, while providing RD communities with tailored, condition-specific knowledge that is often unavailable elsewhere (39, 64, 65). At the same time, this reliance on experiential authority raises questions about whose experiences are mobilized, which voices are amplified, and under what conditions such expertise is recognized by institutional actors.

The present findings add nuance by foregrounding how these activities are grounded in embodied, experiential expertise rather than formal professional authority, aligning with STS accounts of the emergence of “lay experts” in medical contexts (43, 44). From this perspective, knowledge production within RDPAGs is not merely a compensatory response to institutional deficits but a distinct epistemic practice shaped by lived experience, long-term caregiving, and iterative engagement with healthcare systems. At the same time, previous studies caution that the mobilization of experiential expertise is always relational and contingent. As Collins and Evans (45) argue, the legitimacy of non-certified expertise depends on boundary negotiations with professional actors and on the recognition of experience-based knowledge as relevant and credible. Participants’ accounts reflect this tension: while experiential knowledge was sometimes described as opening doors to dialogue and partnership, its acceptance remained uneven and context-dependent.

Secondly, participants portrayed RDPAGs as “knowledge hubs” that seek to stabilize uncertainty for families, particularly in the period immediately following diagnosis. They translate expert knowledge into accessible guides, webinars, and conferences, while facilitating peer-to-peer exchange that allows families to draw on others’ experiences and avoid repeating past mistakes. These initiatives were described as strengthening families’ sense of agency and belonging while helping them navigate complex care systems. Comparable patterns have been documented internationally, where patient organizations are shown to compensate for fragmented care and insufficient professional guidance, particularly in RD contexts (60, 66, 67). Studies from China, Europe, and North America indicate that families often rely on patient organizations as primary sources of practical and interpretive knowledge when formal systems fail to provide coordinated information (38, 68). In addition, organizations offer practical, experience-driven guidance on therapies, diets, rehabilitation, and everyday care, while stepping in with legal support and bureaucratic navigation. Several participants emphasized that this informal support often compensates for gaps in formal systems, such as hospital visits, meal delivery, or assistance with disability appeals, which they perceived as insufficiently responsive (58, 60, 66).

A key outcome of this organizational empowerment is the capacity to produce and disseminate reliable educational resources. Many RDPAGs develop websites, guides, and databases providing information on diseases, treatment options, and expert clinicians, often in collaboration with healthcare professionals, researchers, and industry to ensure accuracy and accessibility (69). They also create supportive communities, address questions left unanswered in clinical settings, and guide families through healthcare systems (11, 39, 41). However, consistent with findings by Wallraf et al. (70) and Dimond and Lewis (60), participants’ accounts also suggest that the production and maintenance of such resources is unevenly distributed and frequently relies on the sustained, often unpaid labour of a small number of highly engaged leaders. This uneven burden raises questions about sustainability and the long-term viability of patient-led knowledge infrastructures in the absence of institutional support.

This dual position, as both affected individuals and knowledge brokers, was particularly evident in how participants described their advocacy work. The metaphor of leaders as “ambassadors” and “advocates” underscores the hybrid nature of their role. Their expertise does not stem from formal training but from personal experience, long-term caregiving, and sustained interaction with healthcare systems. From an STS perspective, this metaphor is analytically productive precisely because it naturalizes authority and representation (71). The process of singularization and mutualization described by Akrich et al. (71) helps explain how RD organizations simultaneously foreground condition-specific expertise while forging alliances around shared systemic problems. This vantage point enables them to mediate between scientific knowledge and the everyday realities of RD care. At the same time, while such ambassadorial roles may grant credibility in professional and policy settings, they may also obscure the contingent, negotiated nature of legitimacy, including tensions around representation, boundary-setting, and emotional labour involved in advocacy. These dynamics raise important questions around positionality, power, and voice in patient-led advocacy, especially when leaders are both caregivers and organizational representatives. Participants’ accounts echo prior observations that experiential expertise can both empower patient organizations and expose them to expectations that exceed their formal capacities (72, 73). These dynamics complicate normative calls for “patient participation,” suggesting that increased involvement without corresponding structural support may risk reproducing inequalities and burnout within advocacy communities.

Thirdly, participants framed RDPAGs as intermediaries seeking to address persistent knowledge gaps among primary care physicians, specialists, and educators, gaps linked to diagnostic delays and inadequate educational support. Through resource development, joint training initiatives, and participation in guideline development, RDPAGs were described as contributing to professional learning around timely diagnosis, patient-centred care, and psychosocial dimensions of RDs (59, 65, 74). These findings align with extensive evidence documenting insufficient RD education among healthcare professionals across Europe and beyond (38, 68). Within this context, patient organizations emerge not as optional partners but as de facto educators operating in the shadow of systemic educational deficits. At the same time, participants’ narratives also highlight the limits of this arrangement, as experiential knowledge is not uniformly welcomed and remains shaped by professional hierarchies and contested boundaries of expertise.

These findings align with broader international evidence showing that insufficient knowledge among healthcare professionals, particularly PCPs, remains a major contributor to diagnostic delays and fragmented care (15–21). In Poland, 95% of students in nursing, physiotherapy, and medicine rated their knowledge as insufficient, and over 90% did not feel prepared to care for RD patients (75, 76). Similar deficits were reported among nurses (77) and physicians in specialization training (17), with comparable findings documented internationally (15, 16, 18–21). Within this context, participants’ accounts illustrate how patient organizations attempt to compensate for systemic educational gaps, while simultaneously highlighting the limits of relying on informal, patient-led knowledge production.

Lastly, participants described public awareness campaigns and media engagement as central strategies for increasing societal recognition of RDs and reducing stigma. They also reported involvement in legislative processes, regulatory review, and the co-development of accessibility and care standards with long-term policy implications. International research similarly documents how patient organizations influence public discourse and policy agendas at national and transnational levels (41, 65). However, both the present findings and prior studies caution that visibility-driven advocacy may privilege positive, mobilizing narratives while rendering less visible experiences of fatigue, conflict, and marginalization within organizations (60, 72).

A significant area of activity involves system-level advocacy—participation in legislative processes, regulatory review, and the co-development of accessibility and care standards with long-term policy implications. This aligns with prior research showing that patient organizations play a key role in shaping public understanding and influencing decision-makers at national, EU, and international levels (69, 78). At the same time, the emphasis on visibility and positive messaging may obscure less publicly shareable experiences of fatigue, conflict, or marginalization, which were largely absent from leaders’ narratives.

Rather than framing institutional recognition and funding as self-evident solutions, these findings suggest the need for a more reflexive approach that considers how patient-led knowledge production is supported, governed, and potentially constrained once incorporated into formal systems. From this perspective, calls for integration are best understood not as normative demands but as empirically grounded responses to the documented reliance of healthcare and education systems on informal, patient-generated expertise.

Beyond the Polish context, the findings also invite reflection on their potential transferability to other national settings. Although this study is situated within the Polish context, similar configurations of patient-led education, advocacy, and informal knowledge brokerage have been documented internationally, particularly in health systems characterized by limited professional training in RDs and fragmented care pathways (38–40, 66, 68, 73). The educational and advocacy roles described here are therefore not unique to Poland but reflect broader patterns observed in RD communities internationally, especially in contexts where institutional knowledge gaps persist. Rather than claiming direct generalizability, this study offers analytically transferable insights into how patient advocacy groups function as informal knowledge brokers across health, education, and policy domains. Readers and policymakers in other settings may assess the relevance of these findings by considering similarities in healthcare organization, professional training, and the role of civil society in RD support. In this way, the study contributes to a growing international understanding of patient-led education and advocacy while remaining grounded in the Polish empirical setting. Seen through an STS lens, RDPAGs emerge not only as advocates but as sites of distributed, negotiated, and sometimes contested knowledge production within contemporary healthcare systems.

At the same time, the findings underscore the epistemic significance and the limits of leadership-based perspectives within RD advocacy. As caregivers and organizational representatives, participants occupied a distinctive position at the intersection of lived experience, collective representation, and system-facing engagement. Their accounts illuminate how experiential knowledge is mobilized and legitimized, while also revealing the tensions, exclusions, and asymmetries that shape patient-led advocacy in practice.

### 4.1 Strengths and limitations

This study has several limitations. Firstly, the small sample size, while sufficient for rich and nuanced thematic development, limits broad generalizability. However, participants were purposefully selected for their leadership experience, enabling informed and reflective accounts. Moreover, as a qualitative study, the design prioritizes interpretive depth over numerical representation. Secondly, the predominance of female participants reflects caregiving dynamics in the RD field, where women often assume informal support roles and carry a disproportionate emotional and mental burden. Nevertheless, this gender imbalance limits the inclusion of more diverse perspectives. Thirdly, the online format may have excluded individuals less engaged in digital spaces, thereby introducing sampling bias.

Fourthly, the single-researcher design may also introduce interpretive bias; however, this aligns with the assumptions of RTA, which recognizes the researcher as an active, reflexive co-constructor of meaning. Reflexivity was maintained throughout the study via memo-writing and critical self-reflection to examine how the researcher’s positionality and assumptions informed theme development. Fifthly, the use of remote interviews limited the ability to observe nonverbal cues and emotional nuance. In addition, one participant provided written responses instead of participating in an interview, which may have limited depth and opportunities for probing. However, the impact of this deviation on the analytic process was mitigated by the limited and supplementary role of this material within the overall dataset.

Sixthly, all participants were organizational leaders of RDPAGs. While this enabled informed, strategic, and reflective accounts, this sampling approach may have introduced a positive bias toward advocacy-oriented interpretations of the role, effectiveness, and impact of RDPAGs, as leaders may occupy relatively privileged or empowered positions within their communities. Their narratives, although deeply informed by personal caregiving and advocacy, may have emphasized organizational achievements, strategic goals, visibility, and system-facing success, while offering more limited insight into everyday challenges, conflicts, disengagement, or unmet needs within patient communities experienced by less visible patient members. Additionally, their dual positionality, as both caregivers and organizational representatives, may have shaped how successes, challenges, and limitations were framed. Future research would benefit from incorporating the perspectives of other stakeholders, including non-leadership patient members, healthcare providers, educators, and policymakers, to triangulate these findings and add further nuance to the understanding of the educational and advocacy roles of RDPAGs, as well as their internal power dynamics, inequalities between patient organizations, and the potential unintended consequences of informal, patient-led knowledge production. Accordingly, the findings should be interpreted as leadership-informed and advocacy-oriented accounts, rather than as exhaustive or representative descriptions of experiences within the broader RD community. Future research should more explicitly examine internal power dynamics, inequalities between patient organizations, and the potential risks and unintended consequences of informal, patient-led knowledge production. These dimensions could not be fully explored within the scope of the present leadership-based study. Lastly, this article reports findings from a single analytic strand, the educational and informational roles of RDPAGs, drawn from a broader study. Other dimensions of patient-led activity, such as emotional support and structural advocacy, will be explored in subsequent analyses.

Despite these limitations, the study fills an important gap in the Polish literature by examining how RDPAGs contribute to RD education and advocacy. The diversity of organizations represented, the richness of narrative accounts, and transparent reporting aligned with COREQ guidelines enhance the credibility and trustworthiness of the findings. Importantly, this research amplifies the voices of those shaping support communities while also highlighting the negotiated, contingent, and sometimes costly nature of patient-led knowledge work.

## 5 Conclusion

This study examined how leaders of RDPAGs in Poland described their educational and advocacy roles within healthcare, education, and policy contexts characterized by limited formal knowledge about RDs. Participants portrayed RDPAGs as actors who translate experiential and scientific knowledge into practical guidance, support families following diagnosis, engage with professionals, and seek to raise public and institutional awareness of RDs.

The findings indicate that, in the absence of comprehensive and coordinated institutional frameworks, healthcare and education systems in Poland already rely substantially on the informal educational and advocacy work of RDPAGs. At the same time, participants’ accounts highlight that these roles are often performed without stable funding, formal recognition, or clearly defined boundaries, and may involve uneven organizational burdens and risks of overextension.

Rather than framing institutional recognition and support as self-evident solutions, this study suggests that closer attention is needed to how patient-led educational and advocacy activities are sustained, governed, and integrated into formal systems. From this perspective, collaboration with RDPAGs may offer important opportunities to address persistent knowledge gaps in RD care, provided that such collaboration is approached reflexively and with sensitivity to issues of sustainability, representation, and role negotiation.

By foregrounding leadership-based perspectives, this study contributes to a growing international literature on patient-led knowledge production and advocacy in RD contexts. While the findings are grounded in the Polish setting, they offer analytically transferable insights into how patient organizations function as informal knowledge brokers across health, education, and policy domains.

Participants’ accounts point to several areas where current reliance on RDPAGs could be made more sustainable, including more structured cross-sector collaboration with professionals, greater stability of funding for educational and support activities, and clearer mechanisms for incorporating experiential knowledge into policy and legislative processes.

## Data Availability

The raw data supporting the conclusions of this article will be made available by the authors, without undue reservation.
