# Supplementary material for: “We are ambassadors, we are advocates”: rare disease patient advocacy groups as knowledge brokers across health and social systems—a qualitative study from Poland
Source: Front Public Health. 2026 Feb 26;14:1743598. doi: 10.3389/fpubh.2026.1743598 (PMC12980798; doi:10.3389/fpubh.2026.1743598)
Supplement: Supplementary file 1 [file Data_Sheet_1.pdf]

Strategies for Building and Sustaining Support Communities of Caregivers of People with Rare Diseases, and the Role of These Communities in Providing Emotional, Informational, and Practical Support

Dear Sir or Madam,

My name is Jan Domaradzki, and I am a researcher at the Department of Social Sciences and Humanities at the Poznan University of Medical Sciences, where I am conducting a study on the role of patient-led networks in rare diseases, specifically, the strategies they adopt in building communities and the mechanisms they use to provide emotional, informational, and practical support. I kindly invite you to participate in an interview to share your personal experiences in supporting the rare disease community.

The study is fully anonymous. I will not ask for any personal data, and the results will be used solely for academic purposes.

Thank you in advance for your support.

With kind regards,

Jan Domaradzki, dr hab., prof. UMP
Department of Social Sciences and Humanities
Poznan University of Medical Sciences
Rokietnicka 7, Poznań
email: jandomar@ump.edu.pl

Participant Consent Form

1. I have been informed about the planned study, including its objectives, purpose, procedures, and methods of data collection.
2. I understand that participation in the study is completely voluntary.
3. I understand that my participation in the study is anonymous.
4. I understand that I may withdraw from the study at any time, without providing any reason, and that doing so will not result in any penalties.
5. I understand that if I have any questions or concerns during the course of the study, I may contact the individuals listed in the study information.
6. I understand that participation in this study is unpaid and does not involve any financial compensation.
7. I confirm that I understand all the information provided to me regarding this study.
8. I voluntarily agree to participate in this study.

□ yes □ no

Participant Demographic Information

1. Age: ______ years

2. Gender:
□ Female □ Male

3. Education:
□ Primary □ Secondary □ Bachelor/Engineer □ Master's degree or higher

4. Position in the organization:
□ President □ Vice-President □ Board Member □ Other (please specify): __________

5. Duration of involvement in the organization:
□ Less than 1 year □ 1–3 years □ 4–6 years □ More than 6 years

6. Size of the organization (number of members or beneficiaries):
□ Up to 50 □ 51–200 □ 201–500 □ More than 500

7. Scope of the organization's activity:
□ Local □ Regional □ National □ International

8. Are you also a parent or caregiver of a person with a rare disease?
□ Yes □ No

1. General Questions

1.1 What were the motivations behind founding the Foundation/Association and its caregiver community?

1.2 How would you describe the current character of the community you have created?

2. Challenges of Caregivers of People with Rare Diseases

2.1 What are the biggest challenges faced by caregivers of people with rare diseases?

2.2 How do these challenges affect the mental health of caregivers?

2.3 How does caregiving affect the quality of life of the entire family?

3. Strategies for Building and Sustaining Communities

3.1 What strategies and actions do caregivers undertake to build and sustain support communities, both in-person and online?

3.2 What tools, platforms, or communication channels are most commonly used to build connections within the community?

3.3 Do you see any differences in the dynamics and functioning of in-person vs. online communities? What are they?

3.4 How do you support community development? Do you organize events, workshops, support groups, or online forums?

3.5 What actions help maintain engagement and cohesion within the community?

4. Types of Support Provided by the Community

4.1 What kind of emotional support does the caregiver community provide? What forms of support are most helpful?

4.2 What kind of informational support (e.g., knowledge about the disease, treatment, care, education) does the community offer?

4.3 What practical forms of support are available (e.g., help with daily tasks, experience sharing)?
4.4 How do you promote awareness about the disease within and outside the community?
4.5 What forms of support do you offer in crisis situations?
4.6 Do you notice differences in the type of support between online and in-person communities?
4.7 What is the importance of experience sharing among caregivers?

5. Role in Clinical Research and Cooperation with Scientists
5.1 What role does the Foundation/Association play in organizing or supporting clinical research and recruiting patients?
5.2 How do you support caregivers in participating in clinical research? What types of support do you offer?
5.3 What is the cooperation like with scientists and research institutions?
5.4 How do you communicate research findings to the community and support ethical conduct in research?

6. Role of Communities and Support Networks in Coping with Challenges
6.1 How does participating in caregiver communities help in coping with difficulties?
6.2 Can you give examples of how building a support network improved caregivers' well-being or quality of life?
6.3 What forms of support do you find most effective in overcoming challenges?
6.4 What social, legislative, educational, or health-related changes have you been able to achieve together?
6.5 How would you assess cooperation with the public sector and the role of associations?

7. Challenges and Needs
7.1 What are the main challenges in creating and sustaining caregiver communities?
7.2 What are the key gaps and needs of the Foundation/Association and caregivers?
7.3 What forms of assistance and support would be most valuable to you in the future?
7.4 What else could help strengthen communities and their positive impact on caregivers and their families?

8. Summary
8.1 What is essential for developing and strengthening the activities of Foundations/Associations and caregiver communities?
8.2 Is there anything you would like to add that has not been covered?
